# Supplementary material for: Dual-comb photothermal spectroscopy
Source: Nat Commun. 2022 Apr 21;13:2181. doi: 10.1038/s41467-022-29865-6 (PMC9023540; doi:10.1038/s41467-022-29865-6)
Supplement: Supplementary file 1 — Supplementary Information [file 41467_2022_29865_MOESM1_ESM.pdf]

Supplementary to
Dual-comb Photothermal Spectroscopy

Qiang Wang[1,2,†,*], Zhen Wang[3,†,*], Hui Zhang[1,2], Shoulin Jiang[4], Yingying Wang[5], Wei Jin[4], and Wei Ren[3,*]

[1]State Key Laboratory of Applied Optics, Changchun Institute of Optics, Fine Mechanics and Physics, Chinese Academy of Sciences, Changchun 130033, China

[2]University of Chinese Academy of Sciences, Beijing 100049, China

[3]Department of Mechanical and Automation Engineering, and Shenzhen Research Institute, The Chinese University of Hong Kong, New Territories, Hong Kong SAR, China

[4]Department of Electrical Engineering, The Hong Kong Polytechnic University, Kowloon, Hong Kong SAR, China.

[5]Institute of Photonics Technology, Jinan University, Guangzhou 510632, China

†These authors contributed equally to this work

*Emails: wangqiang@ciomp.ac.cn; wangzhen@link.cuhk.edu.hk; renwei@mae.cuhk.edu.hk

Supplementary Note 1: Bandwidth of the Fabry-Pérot interferometer

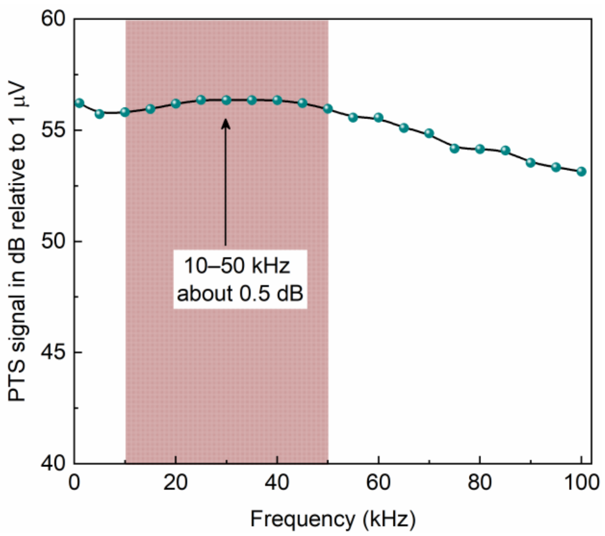

Fig. S1 Evaluation of the bandwidth of the Fabry-Pérot interferometer by measuring the photothermal response of C_2H_2 with a CW pump laser at 1531.6 nm.

The bandwidth of the Fabry-Pérot interferometer (FPI) is evaluated by a standard photothermal test. Instead of the dual-comb source, a CW diode laser at 1531.6 nm is used as the pump for C_2H_2, whereas the probe laser is used the same as that described in the main text. With 2% C_2H_2/N_2 mixture filled in the FPI, the photothermal response is measured by varying the modulation frequency of the pump laser. As shown in Fig. S1, the current FPI shows a relative flat-top with a variation within 3 dB. In this work, we select the band of 10 to 50 kHz, with a variation of 0.5 dB, for multi-heterodyne detection. The response curve in this range can be numerically described by a polynomial fit.

Supplementary Note 2: Phase stabilization of the Fabry-Pérot interferometer

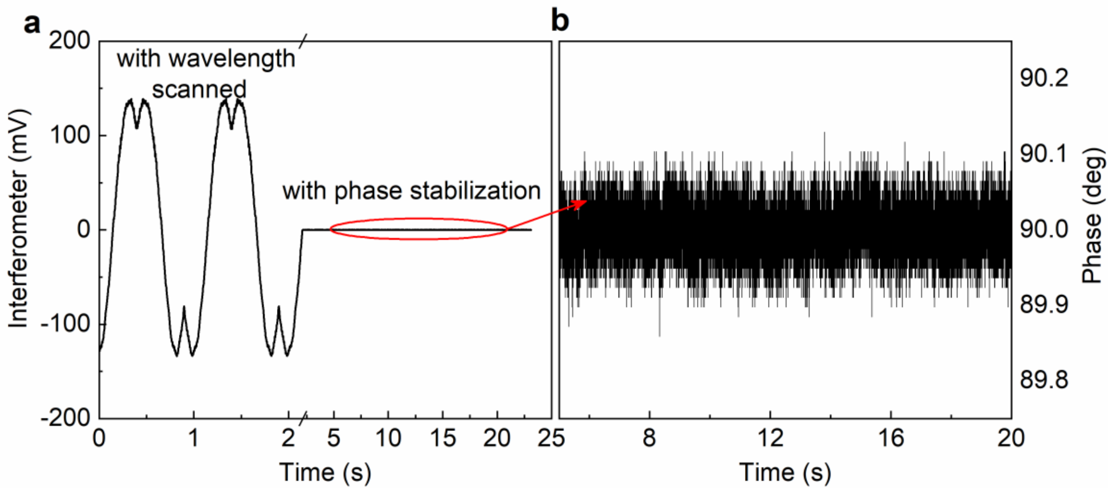

Fig. S2 Phase stabilization of the FPI. a The output of the FPI at the wavelength scanning mode and phase stabilization mode. **b** Evaluation of phase noise after locking the FPI at the quadrature point.

As shown in Fig. 2 of the main text, the FPI output is low-pass filtered and used as the error signal to maintain the FPI operation at the quadrature point. We can get the interference fringe and find the quadrature point by scanning the probe wavelength shown in Fig. S2(a). The probe laser is selected at 1572 nm, corresponding to the non-absorption wavelength and the quadrature point of the FPI. A slow feedback loop of a laser servo (Toptica FALC110) is used in this work for the FPI stabilization. The performance of the FPI stabilization in terms of phase fluctuation at quadrature point is shown in Fig. S2(b). Hence, the FPI shows a phase noise (1σ) of ~1.6′ (0.027°).

Supplementary Note 3: Optical interference effect of the FPI for the pump dual-comb

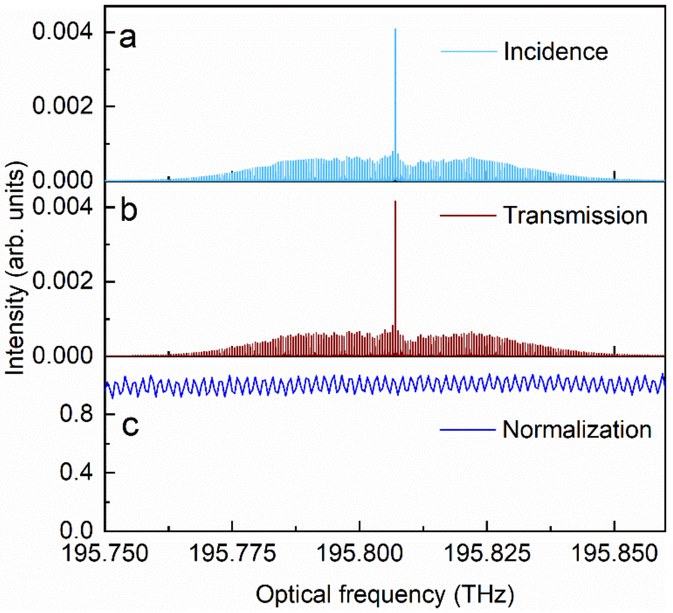

Fig. S3 Optical interference effect of the FPI. a Incident dual-comb spectrum. **b** Transmission

spectrum of the dual-comb through the FPI. **c** Normalization of the transmitted dual-comb spectrum by the incidence.

The dual-comb spectra before and after passing through the FPI are measured and shown in Fig. S3. In this test, the ECDL frequency is tuned to 195.807 THz and the spectral measurement is conducted with an averaging time of 100 s. By dividing the transmission spectrum by the incident one, the slight interference fringe caused by the FPI is observed with an amplitude variation of less than 5%. This effect could be mitigated by adding broadband anti-reflection (AR) coatings for the comb light on the two facets of the single-mode fibers and selecting the probe wavelength beyond the spectral range of the AR coating.
